# Association Between Electronic Health Record–Based Nursing Workload and Turnover: Retrospective Cohort Study

**DOI:** 10.2196/89645

**Published:** 2026-06-12

**Authors:** Linlin Xia, Daphne Lew, Lindsay Tessmer, Elise Eiden, Sunny Lou, Thomas Kannampallil

**Affiliations:** 1 Division of Computational and Data Sciences, Washington University in St. Louis, St. Louis, MO, United States; 2 Institute for Informatics, Data Science and Biostatistics, Washington University School of Medicine in St Louis, St Louis, MO, United States; 3 Barnes-Jewish College, St. Louis, MO, United States; 4 Department of Anesthesiology, Washington University School of Medicine in St. Louis, Washington University in St. Louis, 660 South Euclid Avenue, Campus Box 8054, St. Louis, MO, United States, 1 314-273-7801; 5 Department of Computer Science and Engineering, Washington University in St Louis, , St Louis, MO, United States

**Keywords:** electronic health records, nursing workload, clinical informatics, nurse turnover, workforce retention

## Abstract

**Background:**

Nurse turnover remains a major challenge for health systems, yet objective, scalable measures of workload that predict turnover are limited. Electronic health record (EHR) audit logs offer a potential data source to quantify nursing work patterns.

**Objective:**

This study aimed to evaluate the association between EHR-derived measures of nursing workload and turnover among inpatient nurses.

**Methods:**

We analyzed work-related activities of staff nurses from medical and surgical inpatient units at a large academic medical center from January 1, 2022, to December 31, 2022. Data included deidentified demographics (age, sex, years since licensure, and service group), shift characteristics (number of shifts worked, proportion of night shifts, timing, and location), and measures of work activities derived from EHR audit logs. Audit logs were used to develop measures related to nurse workload, including (1) nurse activities (information review, medication administration, alert management, navigation, documentation, and communication), (2) patient load (based on the number of unique patient charts accessed), and (3) cognitive load (based on the number of patient switches). For nurses who left, we excluded the 6 weeks immediately preceding termination (washout period) and measured workload during the preceding 6-week period; for those who remained, a random 6-week working period was selected. Associations between workload measures and turnover were assessed using mixed-effects logistic regression, adjusting for demographics and shift-related characteristics.

**Results:**

Among 432 nurses (n=363, 84% female; median age 27, IQR 23-36 years), contributing 6812 shifts and approximately 13 million audit log actions, 84 (19%) left the institution in 2022. A higher proportion of medication administration actions was associated with higher odds of turnover (odds ratio [OR] 2.20, 95% CI 1.36-3.54), whereas greater alert engagement (OR 0.48, 95% CI 0.32-0.72) and more years since licensure (OR 0.57, 95% CI 0.38-0.84) were associated with lower odds of turnover.

**Conclusions:**

EHR-derived workload measures, particularly greater medication administration burden and lower alert engagement, were independently associated with the risk of nurse turnover. Patterns of EHR use may help identify nurses at higher risk of leaving an institution and can potentially inform targeted workforce retention strategies.

## Introduction

Nurse turnover has increased exponentially over the past decade, posing a threat to health system stability. In the United States, annual turnover rates rose from approximately 18% in 2016 to approximately 29% immediately following the COVID-19 pandemic [1-3]. Globally, the scale of the problem is equally alarming, with similarly high rates of nurses reporting intentions to leave their organizations [4-6]. High nursing turnover destabilizes care teams, increases medication errors, reduces quality of care, and imposes substantial operational and financial burdens on health care systems [3].

Despite the considerable challenges associated with nurse turnover, its underlying drivers remain poorly understood. Prior work has often linked nurse turnover to factors such as demographics, organizational dynamics [7,8], and workload [1,2,9], primarily relying on observational methods such as surveys, interviews, and focus groups [10-12]. For example, Zheng et al [6] used responses to questionnaires to assess factors such as professional fulfillment, emotional exhaustion, and self-efficacy as contributors to turnover. Other studies have used similar methods to characterize the contributors to nurse turnover and have found high perceived workload, low workplace commitment, poor team collaboration, and lack of recognition from supervisors to be key contributors to nurse burnout and intentions to leave [13,14]. Although these studies provide considerable insights, they depend heavily on self-reported assessments, require significant human effort, and are subject to recall bias, response bias, and limited scalability [1].

Electronic health record (EHR) audit logs offer a scalable approach to quantify nursing workload, capturing time-stamped data on EHR use, including documentation, information review, medication administration, messaging activities, and even indicators of cognitive load such as patient switches and on-screen interruptions [15-17]. However, despite the availability of detailed EHR interaction data from audit logs, little is known about how measures of day-to-day nursing work activities relate to turnover.

In this study, we address this gap by investigating the association between EHR-based measures of nursing workload and subsequent turnover among inpatient nurses at a large academic medical center. By linking nurse work activity metrics with real-world employment outcomes, we aim to provide new insights into how daily clinical work patterns may relate to nurses’ decisions to leave their roles.

## Methods

### Study Setting, Participants, and Inclusion Criteria

This retrospective cohort study included all staff nurses who worked at least 1 clinical shift on a medical or surgical inpatient unit at a large academic medical center in St. Louis, Missouri, between January 1, 2022, and December 31, 2022. Temporary or visiting nursing staff and individuals not officially employed by the hospital were excluded.

### Ethical Considerations

This study was approved by the Washington University Institutional Review Board (IRB#202306138) with a waiver of informed consent. All data were deidentified prior to analysis, and no individually identifiable information was available to the study team. Appropriate measures were taken to protect participant privacy and maintain data confidentiality throughout the study. Participants were not directly involved in the research and did not receive any compensation.

### Data Sources

We obtained deidentified data on eligible nurses from three primary sources: (1) employment records, which provided demographic and employment details (age, sex, years since nursing licensure, duration of employment, and turnover status [ie, whether the nurse left the hospital system in 2022]); (2) nurse shift records, which included daily punch-in and punch-out times, nursing unit (eg, general medicine unit, postprocedure unit, or general surgical unit), level of care (eg, acute, intermediate care, or observation), and clinical service group (medical or surgical); and (3) EHR-based audit logs extracted from Epic (Verona, Wisconsin) Clarity tables (ACCESS_LOG), which provide time-stamped records of nurses’ EHR interactions and the corresponding patient charts.

### Experimental Design

To examine the relationship between EHR-based measures of nurse workload and turnover, we classified nurses into 2 groups: those who left the institution during the 2022 calendar year (“left” group) and those who remained employed throughout the year (“stay” group).

To ensure temporal comparability between groups, we constructed a standardized 6-week observation window for each nurse. The choice of the 6-week period was based on discussions with nursing collaborators across various units rather than on specific empirical evidence.

**Figure 1.**
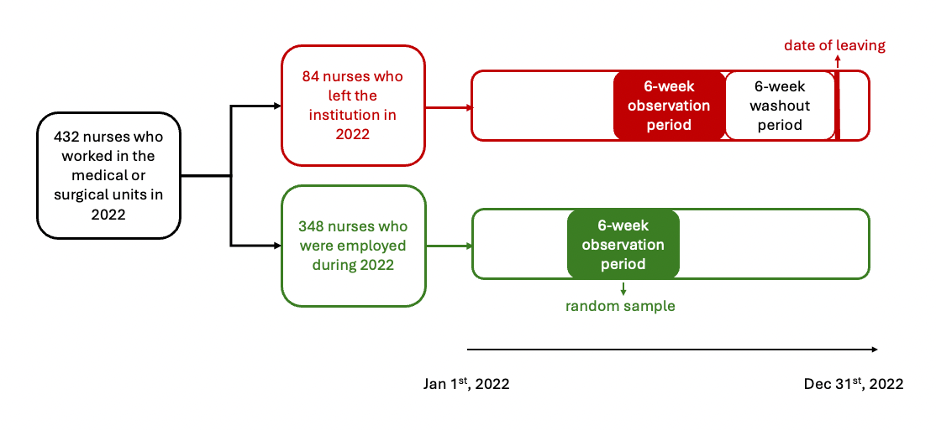
Overall study design. Nurses were grouped into those who were employed at the end of the calendar year (2022) and those who left the institution. For those who left the institution, the date of leaving was used to define a 6-week washout period, and the 6 weeks prior to the washout period were used as the “data period” to calculate the workload metrics. For those who stayed, a random 6-week period during the calendar year was selected for workload metric calculation.

For nurses who left, we imposed a 6-week washout period before their termination date to avoid capturing behavioral changes occurring after the decision to leave. We then analyzed shifts during the 6-week window immediately preceding the washout period (Figure 1; the red vertical bar marks the date the nurse left, and the filled red bar shows the observation window).

For nurses who remained employed, we randomly selected a 6-week working period during the year-long study period (Figure 1; the filled green bar shows the observation window). This approach ensured consistent observation lengths while minimizing temporal bias and reducing the likelihood that observed behaviors reflected impending departure rather than typical workload.

### Data Processing

Each audit log entry included a time stamp, user identifier, patient identifier, and the specific EHR action performed, allowing us to quantify both the frequency and the type of EHR interactions for each nurse during each shift. Audit log events were linked to each nurse’s shift by matching time stamps to staffing punch-in and punch-out records, which defined the temporal boundaries for measurement for each shift. To characterize individual nurse workload patterns, we derived shift-level metrics and then summarized these across shifts within each nurse’s observation window (ie, 6 weeks).

We first used punch-in and punch-out time records to calculate overall schedule-related metrics for each nurse, including the total number of shifts worked during the 6-week observation period and the proportion of shifts occurring during overnight hours (defined as shifts with punch-in or punch-out times between 7 PM and 7 AM; Figures S1 and S2 in Multimedia Appendix 1). These variables were included to adjust for potential differences in workload and turnover risk associated with shift patterns.

Workload metrics included three domains: (1) EHR-based nursing activities, (2) patient load (with the number of unique patient charts accessed per shift used as a proxy), and (3) cognitive effort (with patient-switching actions used as a proxy) [18,19].

To characterize the composition of EHR-based nursing activities, we relied on prior time-and-motion studies that provided detailed insights into nursing workflows in the EHR [20]. The categorization framework was developed through an iterative process involving audit log experts and clinical domain experts (coauthors LT and SL) familiar with clinical and nursing workflows. Audit log actions were initially mapped to functional task types using vendor documentation and institutional data dictionaries and then refined through multiple rounds of structured clinician review to ensure face validity and interpretability.

Specifically, we categorized EHR actions into 6 EHR-based nursing activities: information review (eg, viewing patient reports, storyboard, and clinical notes); medication administration (eg, barcode scanning, barcode processing, and reviewing administration data); documentation (eg, interacting with flow sheets and clinical forms); workflow navigation (eg, accessing the inpatient system list and loading patient lists); active alert management (eg, acknowledging best practice advisories, reviewing medication warnings, or taking recommended actions); and communication (eg, secure chat communication; refer to Table 1 for category descriptions). Table S3 in Multimedia Appendix 1 provides the complete actions of audit log mapping to activity categories.

**Table 1.** Nurse workload categories, their description, and associated example metrics from audit logs^a^.

Categories	Description	Example metrics (from audit logs)
Information review	Encompasses nurse activities involving the examination and evaluation of patient-specific data and associated clinical documentation	Report with patient data viewed Storyboard viewed Flow sheet viewed
Medication administration	Involves clinical workflow and procedural tasks required for the administration of medications	Barcode scanned MAR^b^ barcode processed MAR administration viewed
Documentation	Activities focused on entering, updating, or interacting with clinical documentation	Flow sheet accepted Flow sheet data copied forward Inpatient work list task edited
Navigation	Actions related to moving through the Epic system to access different functions or patient records	Visit a loaded Navigator template Inpatient system list accessed Radar dashboard accessed
Active alerts	Interactions with system-generated alerts and clinical decision support advisories	Best practice advisories acknowledged Medication warning displayed Patient chart advisories viewed
Communication	Activities using secure messaging apps to facilitate communication and care coordination among the health care team	Secure chat conversation opened Secure chat conversation created Secure chat activity accessed

a A full list of the audit logs for each category is provided in Multimedia Appendix 1.

b MAR: Medication Administration Record.

We calculated the number of patient switches per shift as a proxy for cognitive effort, reflecting the frequency with which nurses transitioned between patient charts during active EHR use [15,17]. Consistent with prior work, a patient switch was defined as a transition from one patient’s chart to another within 5 minutes, to reflect active transitions rather than idle time [17].

Additional workload measures included the total number of EHR actions per shift, the number of unique patient charts accessed per shift (ie, patient load), and time spent using the EHR per shift. Time spent using the EHR, measured in minutes, was calculated from audit log data by summing the time intervals between successive audit log actions, excluding intervals exceeding 5 minutes, which were considered periods of inactivity [18,19].

To summarize these workload indicators at the individual nurse level, we aggregated each nurse’s total task-based EHR actions, unique patient charts accessed, and patient switches across the entire 6-week observation period. Specifically, we calculated the proportion of each nurse’s EHR actions falling into each activity category by dividing task-specific action counts by the nurse’s total EHR actions. For shift-level workload frequency measures, we computed the median value of each metric across all observed shifts for each nurse. Together, these task-based EHR activity proportions and shift-level workload frequency measures served as the primary exposure variables, capturing individual differences in overall EHR-based workload.

Covariates in the regression models included demographic characteristics and shift-related patterns. Demographic characteristics included age, sex, years since licensure, and primary clinical service group. Shift-based factors included the total number of shifts worked and the proportion of night shifts.

All data processing and feature engineering were conducted in Python (version 3.9.10; Python Software Foundation) using the following libraries: Pandas (version 2.2.2) for data manipulation, Matplotlib (version 3.10.3) and Seaborn (version 0.13.2) for visualization, and Scikit-learn (version 1.6.1) and Statsmodels (version 0.14.4) for data preparation [21-26].

### Outcome

The primary outcome was nurse turnover, defined as a binary variable indicating whether the nurse left the organization during the 2022 calendar year.

### Statistical Analysis

Descriptive statistics were calculated as medians and IQRs or frequencies and percentages. Univariable comparisons between nurses who stayed and those who left were conducted using the Wilcoxon rank-sum test for continuous variables and the chi-square test for categorical variables. Because task-specific action categories are compositional and collectively sum to approximately 100%, we conducted a collinearity analysis using variance inflation factors to assess potential multicollinearity among these variables (Table S1 in Multimedia Appendix 1). On the basis of this assessment and input from clinician experts, the workflow navigation category demonstrated substantial collinearity with other task categories, particularly information review, and was therefore excluded from multivariable modeling to improve model stability and interpretability; information review was retained given its clinical relevance.

To assess the association between EHR-based nursing workload measures and turnover, we used a mixed-effects logistic regression model with random intercepts for nursing units to account for the contribution of each unit’s culture and work environment to turnover. The statistical model adjusted for demographic factors (age, sex, years since licensure, and service group), shift characteristics (number of shifts worked and proportion of night shifts), and workload intensity (total EHR actions, patient switches, and number of patients cared for). All continuous predictors were standardized using IQR scaling prior to modeling. Thus, reported odds ratios (ORs) and 95% CIs represent the association with a 1-IQR increase (from the 25th to the 75th percentile) in each continuous predictor.

To assess robustness, we conducted a sensitivity analysis using a shorter 2-week washout period prior to each nurse’s departure. Statistical analyses were performed using R (R Foundation for Statistical Computing) with the lme4 package [27].

## Results

### Sample Characteristics

The analytic sample included 432 inpatient nurses who collectively contributed 6812 shifts and more than 13.8 million EHR audit log actions during the study period (Table 2). Of these nurses, 84 (19%) left the institution (Figure 1). The sample was predominantly female (84%), with a median age of 27 (IQR 23-36) years. In total, 60% (n=259) of the nurses worked on surgical services and 40% (n=173) worked on medical services.

**Table 2.** Study cohort characteristics stratified by turnover status (N=432).

Turnover status in 2022	All participating nurses	Nurses who stayed (n=348)	Nurses who left (n=84)	*P* value
Number of shifts	6812	5543	1269	N/A^a^
Number of audit log actions	13,807,571	11,228,809	2,578,762	N/A
Age (years), median (IQR)	27 (23-36)	28 (23-37)	26 (24-31)	.58
Sex, n (%)				.67
Female	364 (84)	295 (85)	69 (82)	
Male	68 (16)	53 (15)	15 (18)	
Clinical service group, n (%)				.60
Surgical unit	259 (60)	206 (59)	53 (63)	
Medicine unit	173 (40)	142 (41)	31 (37)	
Years since licensure, median (IQR)	2 (1-6)	3 (1-9)	1 (1-3)	.01^b^
Shift characteristics, median (IQR)				
Shift counts	17 (12-20)	17 (12-20)	17 (13-19)	.12
Proportion of night shifts (%)	10 (0-94)	11 (0-94)	7 (0-94)	.75
EHR^c^ workload intensity, median (IQR)				
EHR time per shift time (%)	33.87 (28.67-39.09)	33.87 (28.57-39.52)	33.85 (28.93-37.99)	.82
EHR time per shift (minute)	260 (216-302)	261 (215-305)	257 (217-289)	.54
Patient charts accessed per shift	6 (5-9)	6 (5-9)	6 (5-9)	.82
Patient switches per shift	61 (44-82)	60 (44-85)	64 (46-80)	.50
EHR actions per shift	2088 (1464-2723)	2122 (1430-2751)	1964 (1566-2595)	.99
EHR task composition (percentage of total EHR actions), median (IQR)				
Documentation	5.96 (4.75-8.10)	5.91 (4.69-8.15)	6.16 (5.02-7.94)	.38
Medication administration	8.75 (6.65-11.98)	8.54 (6.44-11.69)	9.61 (7.41-12.49)	.02^b^
Navigation	9.41 (5.09-15.50)	9.31 (4.86-15.66)	9.84 (5.71-14.41)	.82
Information review	69.55 (57.30-74.58)	69.58 (57.72-74.84)	69.42 (56.16-74.02)	.67
Active alert	0.16 (0.09-0.27)	0.17 (0.09-0.31)	0.14 (0.06-0.19)	.01^b^
Communication	0.73 (0.36-1.38)	0.71 (0.36-1.35)	0.83 (0.43-1.38)	.31

a N/A: not applicable.

b Indicates statistical significance at the *P*<.05 level.

c EHR: electronic health record.

Nurses who left the organization were younger on average (median 26 vs 28 years), although this difference was not statistically significant. They had substantially fewer years since licensure (median 1, IQR 1‐3 years [Bibr R6]vs median 3, IQR 1‐9 years). Sex distribution and clinical service group were similar between groups.

### Univariable Analysis

Shift patterns did not differ meaningfully between nurses who stayed and those who left, with a comparable number of shifts worked during the observation window (median 17 in both groups) and similar proportions of night shifts.

Overall EHR workload intensity—including median EHR time per shift, proportion of shift time spent in the EHR, total EHR actions, number of unique patient charts accessed, and number of patient switches—was nearly identical between groups (all *P*>.30).

However, 2 EHR task composition measures differed significantly. Nurses who left had a higher proportion of medication administration actions (median 9.61%, IQR 7.41%‐12.49% vs median 8.54%, IQR 6.44%‐11.69%; *P*=.02) and a lower proportion of active alert engagement (median 0.14%, IQR 0.06%‐0.19% vs median 0.17%, IQR 0.09%‐0.31%; *P*=.01). No significant differences were observed across other EHR activity categories, including documentation, information review, navigation, or communication (Table 2).

### Multivariable Analysis

In mixed-effects logistic regression models adjusting for demographic characteristics, shift patterns, and workload intensity, task composition emerged as the primary workload-related predictor of turnover (Table 3). An increase in the proportion of medication administration actions from the 25th percentile (6.65%) to the 75th percentile (11.98%) was associated with more than twice the odds of leaving the institution (adjusted OR 2.20, 95% CI 1.36‐3.54). Conversely, an increase in active alert engagement from the 25th percentile (0.09%) to the 75th percentile (0.27%) was associated with substantially lower odds of turnover (adjusted OR 0.48, 95% CI 0.32‐0.72).

**Table 3.** Multivariable analysis showing the association between nursing workload components, demographics, work-related aspects, and turnover.

Variables	Scaled odds ratio (95% CI)	*P* value
Age (years; as of 2022)	1.04 (0.61‐1.79)	.88
Sex (male vs female)	0.79 (0.39‐1.59)	.50
Clinical service group (surgical vs medicine)	1.05 (0.53‐2.07)	.90
Years since licensure	0.57 (0.38‐0.84)	<.01^a^
Number of shifts worked	0.79 (0.56‐1.12)	.18
Proportion of night shifts	0.89 (0.47‐1.67)	.72
Median EHR^b^ actions per shift	1.43 (0.80‐2.54)	.23
Median patient charts accessed per shift	1.07 (0.87‐1.32)	.53
Median patient switches per shift	0.93 (0.64‐1.35)	.70
Information review (percentage of total EHR actions)	0.93 (0.44‐1.96)	.84
Medication administration (percentage of total EHR actions)	2.20 (1.36‐3.54)	<.01^a^
Documentation (percentage of total EHR actions)	0.95 (0.70‐1.31)	.77
Active alert (percentage of total EHR actions)	0.48 (0.32‐0.72)	<.01^a^
Communication (percentage of total EHR actions)	0.96 (0.61‐1.51)	.86

a Indicates statistical significance at the *P*<.05 level.

b EHR: electronic health record.

Years since licensure also remained significant; an increase from the 25th (1 year) to the 75th percentile (6 years) was associated with lower odds of leaving (adjusted OR 0.57; 95% CI 0.38-0.84). No other demographic variables, shift characteristics, or workload intensity measures demonstrated significant associations with turnover.

### Sensitivity Analysis

A sensitivity analysis using a 2-week washout period before departure produced results consistent with the primary analysis, supporting the robustness of the associations identified (Table S2 in Multimedia Appendix 1).

## Discussion

In this study, we investigated the association between EHR-based nursing workload and the likelihood of leaving the institution. We found that fewer years since nursing licensure and performing a higher fraction of medication administration actions were associated with higher odds of turnover. We also found that an increased fraction of active EHR alerts was associated with lower odds of turnover. Taken together, these findings have implications for understanding and potentially redesigning EHR-based nursing workload.

There are several possible explanations for the finding that a higher fraction of medication administration actions was associated with higher odds of turnover. The association of medication administration and documentation with chronic stress and burden has been well-documented [28]. Medication administration is a complex, multistep process involving identifying patients due for medications, prioritizing schedules, accessing centralized dispensing systems, and individually retrieving medications. These tasks may also include secondary nurse verification or additional preparation steps, most of which are not captured in EHR audit logs. Balancing complex medication schedules and performing detailed documentation can lead to mental fatigue and a persistent sense of “catching up” on charting. Over time, this may contribute to emotional exhaustion and increase the likelihood of turnover intention or actual departure [29,30]. During medication workflows, nurses may also encounter various interruptions—from unit alarms to emergency scenarios and interprofessional requests—which further elevate workload and burden [31]. Additionally, increased medication administration burden may serve as a proxy for higher patient complexity, with sicker patients requiring more frequent interventions, although we could not directly measure patient complexity in this study.

We also found that a higher proportion of active alert engagement was associated with lower odds of turnover. For this study, active alerts were defined as audit log events requiring explicit acknowledgment, often surfacing high-priority safety checks or patient status cues that help structure nurses’ decision-making. Nurses engaged with a median of 1.5 active alerts per shift (IQR 1.0‐3.0), indicating that these events are infrequent and unlikely to represent a significant burden. Rather, they may function as a helpful tool that enhances situational awareness, reinforces clinical priorities, and reduces cognitive load by externalizing key reminders. Therefore, nurses who engage with these alerts may experience greater clarity, support, and alignment with expected workflows, mitigating the sense of chaotic work environments that contribute to turnover. Although we could not differentiate alert types, the small absolute variation in alert fractions across clinicians (0.09% to 0.27%) likely reflects the low frequency of these high-salience events rather than differences in overall workload. Differences in alert engagement may signal how effectively nurses interact with digital workflow supports. Importantly, our model adjusted for demographics, shift type, clinical service, and overall EHR activity, making it less likely that this association reflects underlying workload differences alone. Future work characterizing alert content, cognitive load, and perceived usefulness may clarify whether active alert engagement functions as an indicator of supportive digital environments that promote clinician engagement and retention.

This study has several strengths. It was a longitudinal study that included all nurses at a large academic medical center over 1 year (432 nurses, 6812 shifts, >13 million audit log actions), providing a detailed and objective assessment of EHR-based workload. Our audit log measures captured granular shift-level behaviors, offering a scalable framework to quantify workload patterns that may influence clinician retention. Consistent with prior time-and-motion studies, we found that nurses spent approximately 34% of each shift on EHR activities, including documentation, chart review, and medication management, highlighting the substantial cognitive and administrative demands of modern nursing work [20].

This study also has several limitations. First, this was a single-center study of specific inpatient settings over 1 year. Although we had a large sample, findings may not generalize to other institutions or regions. In addition, the cohort was relatively young (median age 27 years) and in an early career stage (median 2 years since licensure), which may not reflect the broader nursing workforce. Turnover drivers in this population may differ from those in more experienced nurses (eg, career development vs long-term occupational strain), which may influence the observed associations.

Second, audit logs do not capture all aspects of nursing work—such as direct patient care, interpersonal interactions, or work with other technologies (eg, medication-dispensing systems). Thus, our workload measures reflect EHR-based workload only. However, prior research demonstrates that audit logs offer an objective measure of cognitive and administrative workload, capturing a critical aspect of modern clinical practice that can influence clinician engagement and retention [15,32].

Third, our dataset was limited to nurses directly employed by the institution; in other words, the service time only reflected their tenure as employees. If a contingent or agency nurse transitioned to direct employment, their prior service as a nonemployee was not reflected. Furthermore, agency or travel nurses who were not direct employees of the health system were not included in our sample.

Fourth, patient complexity and exact nurse-patient assignments could not be fully accounted for. Patient load was derived from EHR audit logs and operationalized as the number of unique patient charts accessed per nurse, rather than staffing assignment data. Although we adjusted for patient load using this measure and included unit type as a covariate, these proxies may not fully capture patient complexity or staffing patterns. Because nurses may access charts for patients who are not formally assigned to them, this measure may not precisely reflect true patient assignments, introducing potential measurement imprecision. Medication administration–related EHR activity may reflect higher patient complexity, medication burden, or assignment to higher-intensity units rather than workload strain alone, and residual confounding by unmeasured clinical factors (eg, patient acuity) remains possible. Direct measures of patient acuity (eg, case-mix index or high-risk medication exposure) were not available in this dataset.

Fifth, our analysis focused on nurses who fully left the organization and did not include those who transferred internally to other units. To avoid mixing data from prior assignments, our observation window was restricted to each nurse’s current unit. Although internal transfers were relatively few, they may still create administrative burden, incur training costs, and pose temporary staffing challenges, which warrants further study alongside complete organizational turnover. Additionally, turnover was defined based on recorded separation from employment, and the reason for leaving (eg, voluntary resignation, termination, or contract completion) was not available, which may introduce heterogeneity in the outcome.

Sixth, workload metrics were aggregated at the nurse level using medians and proportions across shifts to align exposure measurement with a nurse-level outcome of turnover. Although this approach reduces noise in shift-to-shift variability and reflects overall workload exposure during the observation period, it may obscure within-nurse variability, including high-burden shifts. As such, infrequent but extreme workload experiences may not be fully captured. Multilevel shift-based modeling could potentially help in characterizing such patterns.

Finally, we applied a 6-week washout period, based on input from clinical collaborators, to account for the time nurses typically take to decide to leave the organization. We acknowledge that individual decision timelines vary; however, sensitivity analyses using a shorter, 2-week washout period confirmed the robustness of our findings.

Despite these limitations, this study demonstrates a scalable, data-driven approach to quantifying EHR-based nursing workload and its association with turnover. Findings highlight the potential of granular workload measures to identify patterns of digital work allocation that may relate to clinician retention. Future work should integrate direct measures of patient complexity and qualitative assessments of unit culture, interpersonal dynamics, and institutional factors, as well as validate these measures across diverse health systems, to better understand how digital workload interfaces with nurse engagement, burnout, and workforce stability.

## Supplementary material

10.2196/89645 Multimedia Appendix 1 Association between electronic health record–based nursing workload and turnover.

## Abbreviations

EHR: electronic health record
OR: odds ratio
