# Supplementary material for: Association Between Electronic Health Record–Based Nursing Workload and Turnover: Retrospective Cohort Study
Source: JMIR Nurs. 2026 Jun 12;9:e89645. doi: 10.2196/89645 (PMC13263017; doi:10.2196/89645)
Supplement: Multimedia Appendix 1 [file nursing-v9-e89645-s001.docx]

Association Between EHR-based Nursing Workload and Turnover

^1^Linlin Xia, MS*, ^2^Daphne Lew, PhD MPH*, ^3^Lindsay Tessmer, RN BSN, ^4^Elise Eiden, MS, ^4^Sunny Lou, MD PhD**, ^4,5,6^Thomas Kannampallil, PhD**

**Supplemental Figure 1. Distribution of Punch-In Records Across All Nurse Shifts.**


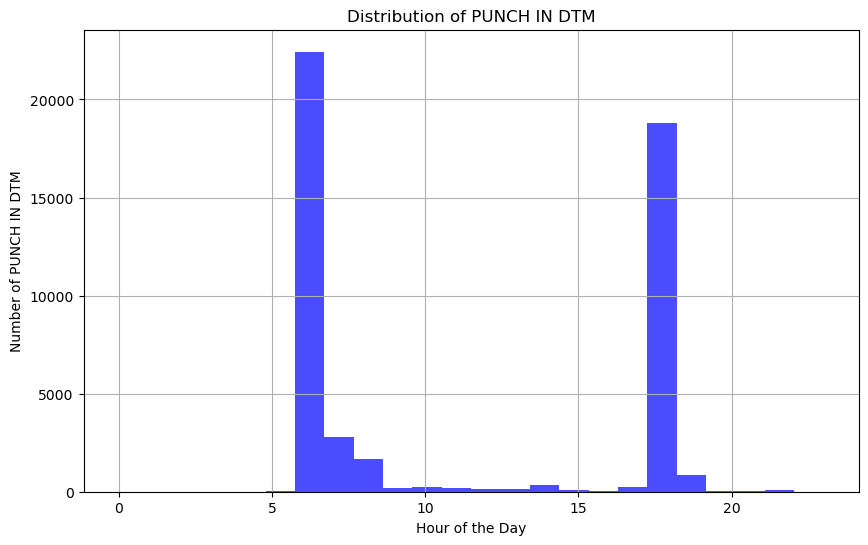


**Supplemental Figure 2. Distribution of Punch-Out Records Across All Nurse Shifts.**


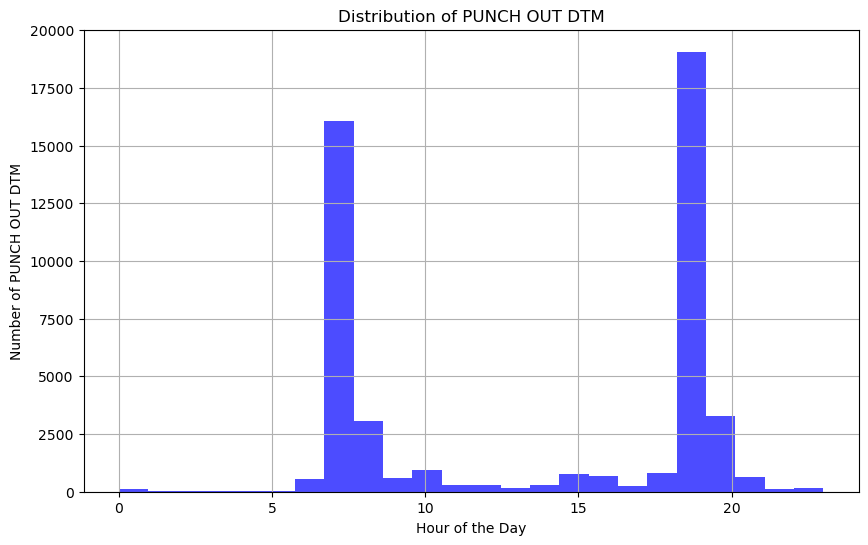


**Supplemental Table 1. Variance Inflation Factors for Predictors Included in the Model.**

| **Variable** | **VIF** |
| --- | --- |
| Sex (Male vs Female) | 1.047577 |
| Years Since Licensure | 3.120041 |
| Age (as of 2022) | 3.333486 |
| Clinical Service Groups (Surgical vs Medicine) | 1.266284 |
| Number of Shifts Worked | 1.070857 |
| Patient Switches per Shift | 2.190956 |
| Patient Charts Accessed per Shift | 1.574281 |
| EHR Actions per Shift | 2.309342 |
| Proportion of Night Shifts | 1.310037 |
| Active Alert | 1.16998 |
| Communication | 1.742511 |
| Documentation | 2.539162 |
| Information Review | 13.51382 |
| Medication Administration | 4.140071 |
| Navigation | 7.056922 |

**Supplemental Table 2. Multivariable Analysis of Associations Between Nursing Workload, Demographics, Work Factors, and Turnover (Two-Week Washout Period; Sensitivity Analysis).**

| **Variable** | **Scaled OR (95% CI)** | **P-value** |
| --- | --- | --- |
| Age (as of 2022) | 1.31 (0.76 – 2.26) | 0.33 |
| Sex (Male vs Female) | 0.58 (0.29 – 1.16) | 0.13 |
| Clinical Service Groups (Surgical vs Medicine) | 0.78 (0.41 – 1.48) | 0.44 |
| Years Since Licensure | 0.50 (0.35 – 0.72) | < 0.01** |
| Number of Shifts Worked | 0.39 (0.27 – 0.56) | < 0.01** |
| Proportion of Night Shifts | 1.36 (0.74 – 2.51) | 0.32 |
| Median EHR Actions per Shift | 1.35 (0.79 – 2.33) | 0.27 |
| Median Patient Charts Accessed per Shift | 1.13 (0.89 – 1.43) | 0.32 |
| Median Patient Switches per Shift | 1.04 (0.76 – 1.43) | 0.80 |
| Information Review (% of Total EHR Actions) | 0.82 (0.42 – 1.58) | 0.55 |
| Medication Administration (% of Total EHR Actions) | 1.96 (1.33 – 2.88) | < 0.01** |
| Documentation (% of Total EHR Actions) | 1.08 (0.81 – 1.43) | 0.60 |
| Active Alert (% of Total EHR Actions) | 0.55 (0.40 – 0.76) | < 0.01** |
| Communication (% of Total EHR Actions) | 1.40 (1.00 – 1.96) | 0.05 |

**Supplemental Table 3. Mapping of Audit Log Metrics to Activity Categories.**

| **Metric ID** | **Action Description** | **Activity Category** |
| --- | --- | --- |
| 17380 | Automatic actions performed by a BestPractice Advisory | Active Alerts |
| 17338 | BestPractice Advisories displayed | Active Alerts |
| 17336 | BestPractice Advisories viewed | Active Alerts |
| 19125 | Patient Chart Advisories viewed | Active Alerts |
| 17381 | BestPractice Advisories acknowledged | Active Alerts |
| 17333 | Actions taken from a BestPractice Advisory | Active Alerts |
| 34097 | Secure chat conversation opened | Communication |
| 34098 | Secure chat conversation created | Communication |
| 20008 | In Basket message created | Communication |
| 34099 | Secure chat activity accessed | Communication |
| 34707 | Flowsheet accepted | Documentation |
| 34708 | Flowsheet data copied forward | Documentation |
| 34175 | Inpatient Work List task edited | Documentation |
| 34100 | Inpatient Care Plan activity accessed | Documentation |
| 1226 | Edit AVS navigator accessed | Documentation |
| 60012 | Clinical Note Signed | Documentation |
| 34720 | LDA Avatar accessed | Documentation |
| 34014 | Care Plan progress modified | Documentation |
| 34167 | Patient Education documented on | Documentation |
| 34700 | LDA properties accessed | Documentation |
| 34701 | LDA properties accepted | Documentation |
| 17213 | Pharmacy for encounter modified | Documentation |
| 17278 | Order Review activity accessed | Documentation |
| 34012 | Care Plan modified | Documentation |
| 34704 | Intake/Output activity accessed | Documentation |
| 34176 | Inpatient Work List task added | Documentation |
| 60010 | Pend clinical note | Documentation |
| 34163 | Patient Education resolved | Documentation |
| 49001 | ED Visit Navigator Arrival section accessed | Documentation |
| 2343 | Consents navigator section accessed | Documentation |
| 34172 | Patient Education assessment filed | Documentation |
| 15136 | Registration/ADT workflow initiated | Documentation |
| 15137 | Registration/ADT workflow finished | Documentation |
| 15629 | Patient communication preferences subcomponent | Documentation |
| 17008 | Report with patient data viewed | Information Review |
| 20607 | Storyboard viewed | Information Review |
| 17625 | FYI activity accessed | Information Review |
| 17626 | FYI activity exited | Information Review |
| 34706 | Flowsheet viewed | Information Review |
| 34005 | Notes viewed | Information Review |
| 17256 | Results Review accessed | Information Review |
| 17007 | Report viewed for an order | Information Review |
| 49017 | Visit Navigator Flowsheet section viewed | Information Review |
| 17258 | Results Review exited | Information Review |
| 34137 | Patient Education information viewed | Information Review |
| 20002 | HTML table viewed | Information Review |
| 17000 | Chart Review Encounters tab selected | Information Review |
| 15604 | Patient Emergency contacts grid accessed | Information Review |
| 34128 | Patient device data accessed | Information Review |
| 17227 | Chart Review Note report viewed | Information Review |
| 94023 | Report viewed | Information Review |
| 94009 | Patient photo viewed | Information Review |
| 19120 | Identity report accessed | Information Review |
| 17228 | Chart Review Encounter report viewed | Information Review |
| 17245 | Demographics viewed | Information Review |
| 35015 | Detailed report viewed in Reporting Workbench | Information Review |
| 15047 | Patient Address subcomponent accessed | Information Review |
| 15048 | Patient Demographics subcomponent accessed | Information Review |
| 15651 | Form viewed | Information Review |
| 17027 | Chart Review Notes tab selected | Information Review |
| 17012 | Chart Review Other Orders tab selected | Information Review |
| 17234 | Chart Review Order report viewed | Information Review |
| 19019 | Potential Duplicates checked | Information Review |
| 17821 | Care Teams viewed | Information Review |
| 17822 | Care Teams exited | Information Review |
| 94019 | Allergies viewed | Information Review |
| 17233 | Chart Review Media report viewed | Information Review |
| 17904 | SmartForm viewed | Information Review |
| 20003 | App Report Viewed | Information Review |
| 34200 | Barcode scanned | Medication Administration |
| 34155 | MAR barcode processed | Medication Administration |
| 34146 | MAR administration viewed | Medication Administration |
| 34142 | MAR administration accepted | Medication Administration |
| 34141 | MAR accessed | Medication Administration |
| 17108 | Order list changed | Medication Administration |
| 34154 | MAR admin stopped opening | Medication Administration |
| 34143 | MAR administration aborted | Medication Administration |
| 34152 | MAR administration edited | Medication Administration |
| 86350 | Rover barcode scanned | Medication Administration |
| 86355 | Administration accessed in Rover | Medication Administration |
| 34140 | Inpatient system list accessed | Workflow Navigation |
| 34161 | Inpatient Patient Lists loaded | Workflow Navigation |
| 20620 | Visit Navigator template loaded | Workflow Navigation |
| 33500 | Radar Dashboard accessed | Workflow Navigation |
| 14070 | User authenticated | Workflow Navigation |
| 24055 | Unit Manager accessed | Workflow Navigation |
| 34116 | Inpatient Work List viewed | Workflow Navigation |
| 34245 | Sign In viewed | Workflow Navigation |
| 34246 | Sign In modified | Workflow Navigation |
| 17824 | Care Teams modified | Workflow Navigation |
| 17215 | Release Orders activity accessed | Workflow Navigation |
| 34162 | Inpatient Patient Lists search | Workflow Navigation |
| 19041 | Patient selected from lookup | Workflow Navigation |
| 94500 | Mobile session created | Workflow Navigation |
| 17288 | Schedule loaded | Workflow Navigation |
| 94400 | Rover login success | Workflow Navigation |
| 19040 | Patient Lookup search | Workflow Navigation |
| 24550 | Patient Station accessed | Workflow Navigation |
| 17210 | Child order released | Workflow Navigation |
| 94027 | Mobile platform opened patient | Workflow Navigation |
| 14011 | Login failed | Workflow Navigation |
